# Fluorescent Probes Design Strategies for Imaging Mitochondria and Lysosomes

**DOI:** 10.3389/fphar.2022.915609

**Published:** 2022-07-19

**Authors:** Huimin Chen, Zhenjie Yu, Shiwei Ren, Yuyu Qiu

**Affiliations:** ^1^ Institute of Materia Medica, Science and Technology Innovation Center, Shandong First Medical University and Shandong Academy of Medical Sciences, Jinan, China; ^2^ Department of Biochemistry, Shandong First Medical University and Shandong Academy of Medical Sciences, Tai’an, China

**Keywords:** lysosomes, mitochondria, small-molecule fluorescent probe, interaction, imaging

## Abstract

Modern cellular biology faces several major obstacles, such as the determination of the concentration of active sites corresponding to chemical substances. In recent years, the popular small-molecule fluorescent probes have completely changed the understanding of cellular biology through their high sensitivity toward specific substances in various organisms. Mitochondria and lysosomes are significant organelles in various organisms, and their interaction is closely related to the development of various diseases. The investigation of their structure and function has gathered tremendous attention from biologists. The advanced nanoscopic technologies have replaced the diffraction-limited conventional imaging techniques and have been developed to explore the unknown aspects of mitochondria and lysosomes with a sub-diffraction resolution. Recent progress in this field has yielded several excellent mitochondria- and lysosome-targeted fluorescent probes, some of which have demonstrated significant biological applications. Herein, we review studies that have been carried out to date and suggest future research directions that will harness the considerable potential of mitochondria- and lysosome-targeted fluorescent probes.

## 1 Introduction

Mitochondria, popularly termed the cellular “powerhouses,” are one of the most significant constituents of eukaryotic cells. The mitochondria not only plays an important role in adenosine triphosphate (ATP) production but also performs numerous essential functions within the cells, such as transmission of information, induction of cell differentiation, growth, and apoptosis (Vakifahmetoglu-Norberg et al., 2017). Mitochondria have a distinctive double-membrane structure, playing important roles in their unique and complicated functions. Lysosomes are “digestion workshops” in cells, participating in the apoptotic process and other types of cell death (Luzio et al., 2007). They can function alone to participate in normal biological processes and can also interact with each other to accelerate the transmission of materials and communication with the external environment, which allows cells to improve their biological functions.

Apart from playing their own distinctive function, mitochondria could interact with lysosomes. At present, the interactions between mitochondria and lysosomes have been extensively explored, such as the fusion of the lysosomes with mitochondria during the process of autophagy (Chen et al., 2019a) and the mitochondrial and lysosomal contact (MLC) (Wong et al., 2019). Their contact promotes transmission of materials and information. When their contacting function is disrupted, the occurrence of human-related diseases, such as Parkinson’s and lysosomal storage–related diseases, is noted (Wong et al., 2019). Therefore, the timely monitoring of their dynamic changes and the estimation of the reactive small-molecule (RSM) levels are very important for identifying their physiological function and the pathogenesis of the related diseases. Several RSMs have been found in the mitochondria and various enzymes exist in the lysosome, whose dysfunction can participate in the progression of human-related diseases, such as metabolic diseases, heart failure, neurodegenerative diseases (Marc et al., 2009), Alzheimer’s disease (Nixon et al., 2000), and Parkinson disease (Daniel et al., 2008).

Fluorescence microscopy (FM) is a powerful tool for studying cellular dynamics, which has been used widely to study the interaction mechanism between mitochondria and lysosomes (Chen et al., 2019b; Chen et al., 2020). However, among imaging organelle interaction, the systematic introduction of mitochondrial and lysosomal imaging strategy is rare. Therefore, the present review focuses on some new imaging strategies of fluorescence probes targeting mitochondria and lysosomes.

## 2 Fluorescence Probes Design Strategies for Mitochondria Imaging

**FIGURE 1:**
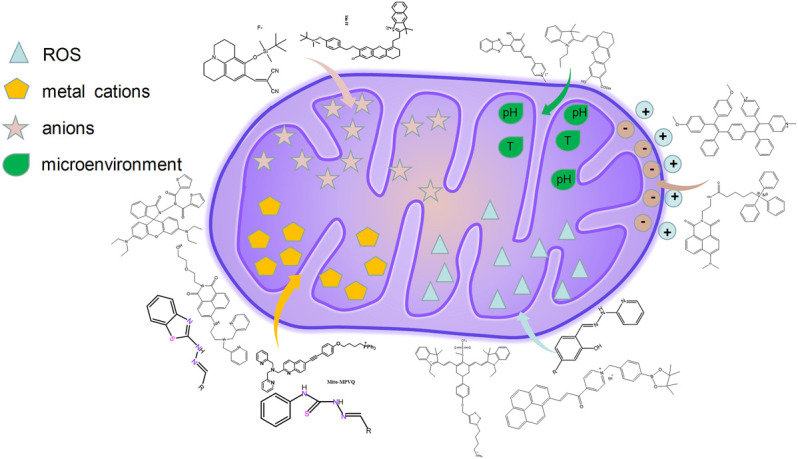
The design strategies of fluorescence probes targeting mitochondria.

Small-molecule fluorescent probes targeting mitochondria can enable the imaging of these organelles to detect the dynamic location and morphological changes of the mitochondria and observe their physiological process (Wei et al., 2022). The fluorescent probes used for imaging mitochondria are mainly divided into the following two types: bearing unique positive charge and targeting the contents of mitochondria and lysosomes. Hence, membrane permeable cationic compounds could be enriched into mitochondria because of their electrophoresis effect. The commercial probe targeting mitochondria, MitoTracker Green (MTG) with positive charge, has been widely used to target mitochondria selectively. In addition, triphenylphosphonium (TPP) with large hydrophobic radius has also been used as a targeting group for mitochondria owing to its high membrane permeability (Figure 1) (Tian et al., 2022).

### 2.1 Single Functional Fluorescent Probes Targeting Mitochondria *via* Positive Charge

Mitochondrial membrane potential is the main component of proton motion dynamics, which is formed by protons pumped from the mitochondrial matrix to membrane. The mitochondria keep the negative transmembrane potential up to −180 mV if the potential in the cytoplasm is 0 mV (Tian et al., 2022). Based on these characterizations, positive charge has been widely applied to target mitochondria.

**FIGURE 2:**
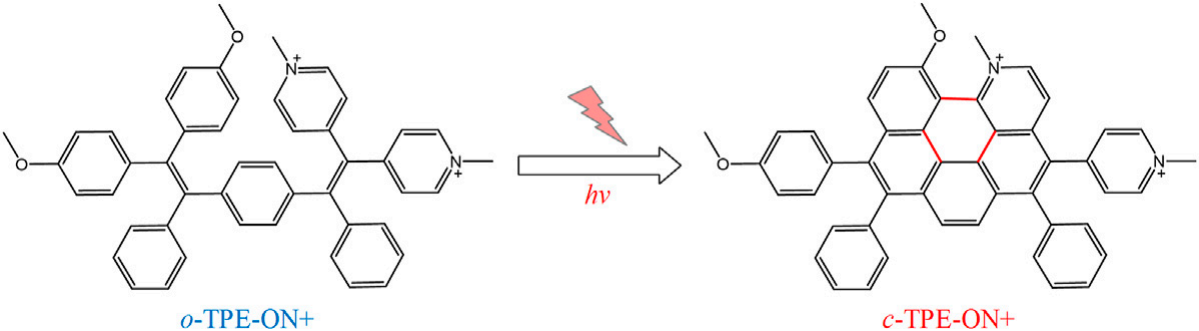
The photocyclodehydrogenation process of ***o*-TPE-ON+** (Gu et al., 2016)

Recently, Gu et al. have designed a novel photoactivatable bio-probe, ***o*-TPE-ON+** (Gu et al., 2016), which can indicate the characterization of spontaneous scintillation without any imaging buffer or additives. Interestingly, this probe can target the mitochondria specifically, which is probably owing to the accumulation of negative charge of mitochondrial outer membrane. In addition, ***o*-TPE-ON+** has been thought as a better choice for fluorescence imaging on the basis of its better cell permeability and outstanding biocompatibility. And the probe ***o*-TPE-ON+** has tracked the high-resolution nanoscopic imaging and dynamic changes of the mitochondria (Gu et al., 2016). Huimin et al. (2022) utilized Cy5 to link with dextran to target and visualize the mitochondrial changes under dextran, which realizes the drug-visualized study at the organelle scale. Saipeng et al. (2015) also designed a mitochondrial fluorescence probe, **NPA-TPP**, with a fluorescent group of 1,8-naphthalimide group and the targeting group of TPP (Figure 2).

### 2.2 Fluorescent Probes Detecting Mitochondrial Contents

It has been demonstrated that there are several RSMs in the mitochondria. The RSMs mainly include reactive oxygen species (ROS), active nitrogen, metal cations, protons, and anions. Therefore, to visualize the distribution and action mechanism of RSMs in the mitochondria, various researchers have designed and synthesized several small-molecule fluorescent probes that target specific mitochondrial contents to further study the action targets and mechanism of drugs, which provides a powerful tool for the integration of diagnosis and treatment of diseases (Liu et al., 2021; Huimin et al., 2022).

#### 2.2.1 Application of the Fluorescent Probes in the Detection of Reactive Oxygen Species in Mitochondria

The survival of cells depends largely on mitochondrial function, which was recognized as an important target for potential drug development (Bras et al., 2005). In recent years, the mitochondria has been recognized as an important target of many drugs. It has been reported that one of the main ways of causing mitochondrial damage is the abnormal level of intracellular ROS. The ROS includes peroxides, super oxides, hydroxyl radicals, singlet oxygen, etc.

**FIGURE 3:**
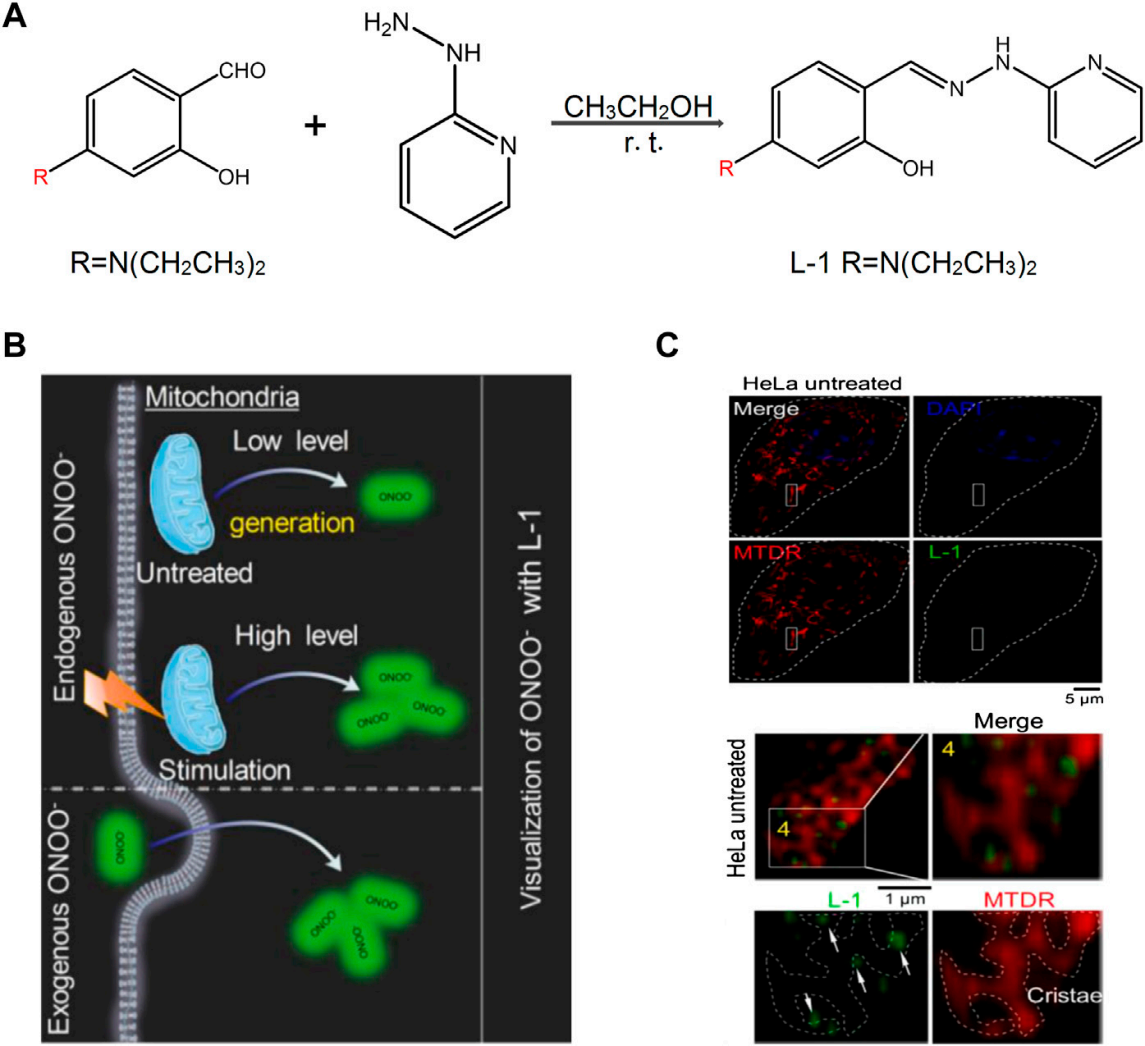
The design of **L-1** and its response with ONOO^−^ (Liu et al., 2021). **(A)** Synthetic route of probe **L-1**. **(B)** Proposed ONOO^**−**^ visualization mechanisms for probe **L-1** in living cells. **(C)** Super-resolution visualization of ONOO^−^ using **L-1** in living cells.

Among them, peroxynitrite (ONOO^−^) is a major one because of its function of signal transduction and antibacterial activities in the biosystems (Radi, 2013). To detect ONOO^−^ selectively, Liu et al. (2021) designed a probe **L-1**, a “landmine,” to monitor the ONOO^−^ levels in living cells with higher selectivity. In addition, they proposed a novel strategy “landmine warfare strategy.” “Landmine” **L-1** without fluorescence was distributed evenly in the cell matrix and could release fluorophore when “engineer” ONOO^−^ was generated in the mitochondria (Figure 3). Under SIM, it could be found that the “engineer” ONOO^−^ acted alone at the mitochondrial cristae and emitted fluorescence. The “landmine warfare strategy” provided a novel designed method for innovative drugs development of diseases which resulted owing to the abnormal ONOO^−^ concentration and provided a novel research direction for other ROS materials in the mitochondria. H_2_O_2_ is one of the major forms of ROS, which participates in the process of a cell’s growth metabolism and energy production (Chen et al., 2014; Kangnan et al., 2020a). Kangnan et al. (2020b) designed a TP probe, **Pyp-B**, to detect the concentration of H_2_O_2_ in the mitochondria. Xiaoyue et al. (2018) developed the near-infrared fluorescent probe **Mito-Cy-Tfs** to detect the level of superoxide anion (O_2_^•−^) and the relationship between O_2_^•−^ concentration and apoptosis during ischemia/reperfusion.

#### 2.2.2 Fluorescent Probe Detection of Metal Cations in Mitochondria

**FIGURE 4:**
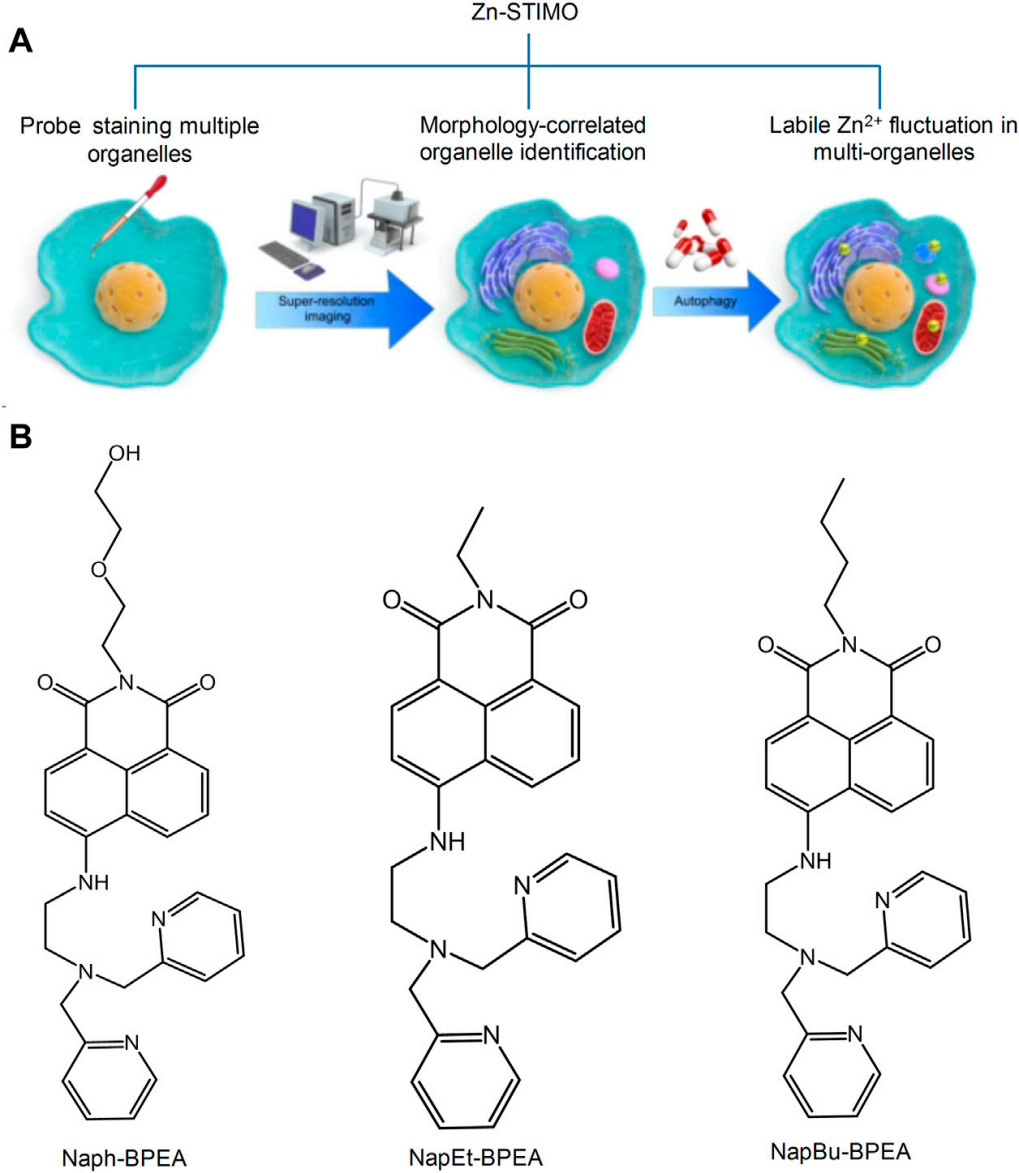
Schematic illustration of Zn-STIMO, and the design of probe candidate for Zn-STIMO (Fang et al., 2021). **(A)** The scheme of Zn-STIMO; **(B)** the structure of fluorescent probes targeting Zn^2+^, **Naph-BPEA**, **NapEt-BPEA**, and **NapBu-BPEA**.

Metal ions are required for mitochondrial physiology in many aspects. Copper, iron, manganese, and zinc play an important role in organ metalloenzymes and metalloproteins (Pierrel et al., 2007). In biological systems, the zinc ion (Zn^2+^) mainly participates in certain life processes, such as DNA synthesis, enzyme catalysis, and gene transcription. In recent years, a large number of evidence have proved that Zn^2+^ is essential to the autophagy process, and autophagy can promote large changes of Zn^2+^. Therefore, the detection of the levels of Zn^2+^ has become one of the research hotspots of mitochondrial fluorescent probes. In recent years, researchers have designed and synthesized various fluorescent probes for the intracellular detection of Zn^2+^ in the cells; however, certain probes cannot target the mitochondria (Hung et al., 2013; Liuzzi and Yoo, 2013; Ding and Zhong, 2017). To determine the importance of Zn^2+^ in autophagy and signal transformation, Fang’s group developed a series of probe targeting Zn^2+^ in many organelles simultaneously and proposed a novel concept, Zn-STIMO, of tracking Zn^2+^ in multiple organelles (Figure 4) (Fang et al., 2021). They found that mitochondrial autophagy inducer CCCP-induced mitophagy in HeLa cells is associated with unstable Zn^2+^ enhancement. The results showed that SIM technology would become a reliable tool detecting unstable Zn^2+^, which also demonstrated that the organelle identification related with super resolution morphological study would have an amazing potential in tracking the biological species and events of specific organelles in organoids. Ning et al. (2016) developed a two-photon ratio probe (**Mito-MPVQ**) targeting the mitochondria to detect Zn^2+^ levels. Triphenylphosphine was used as the targeting group of the probe. Following the attachment of a fluorescent group, this probe was localized in the mitochondria and improved the two-photon signal detection for Zn^2+^.

In addition, during these years, previous researchers have designed and synthesized several fluorescent probes to detect other metal cations in the mitochondria. For example, Wang et al. (2018) designed the mitochondria-targeting fluorescent probe (**PyCM-2**) and (**PyCM-3**) to detect Au^3+^ levels.

#### 2.2.3 Fluorescent Probe Detection of Anions in Mitochondria

**FIGURE 5:**
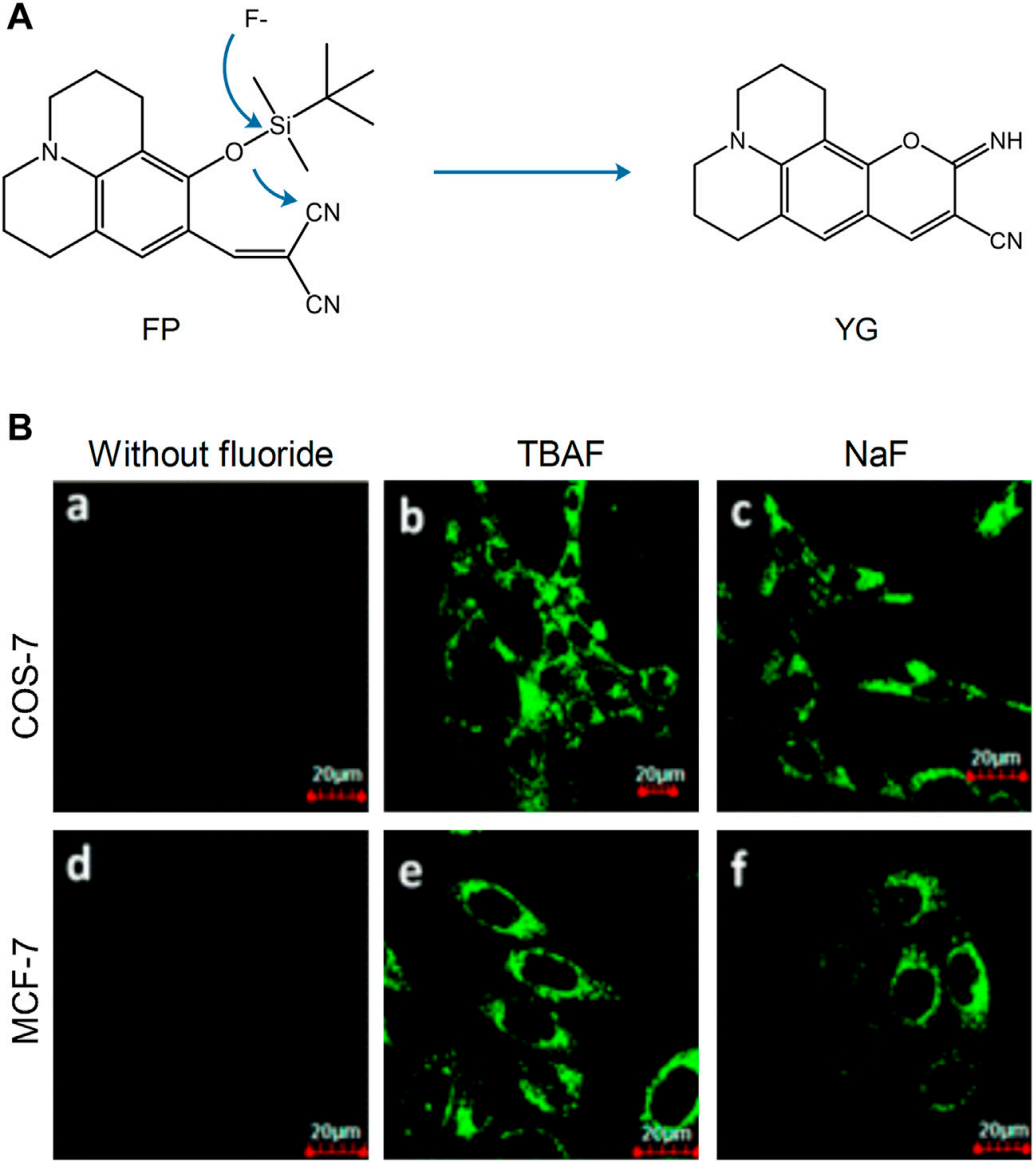
The reaction mechanism and fluorescence imaging of probe **FP** (Shiling et al., 2014). **(A)** Proposed reaction mechanism of **FP**. **(B)** Fluorescence imaging of COS-7 and MCF-7 cells incubated with probe **FP** (2.5 μM) before (a and d) and after (b, c, e, and f) being treated with TBAF, NaF (100 μM).

Although the detection of cations in the mitochondria has been extensively examined, the role of anions is also very important. Fluorine ions can cause metabolic diseases following their accumulation in the mitochondria. In 2014, Shiling et al. designed and synthesized the mitochondrial fluorescent probe **FP**, which was used to detect fluorine (Shiling et al., 2014). This probe was localized in the mitochondria. The fluoride ions could knock out the silyl protecting group, and subsequently the phenoxide reacted with the −CN group, which induced nonfluorescent **FP** to highly fluorescent **YG** and emitted strong green fluorescence (Figure 5). Therefore, **FP** was used to detect and image fluorine in the mitochondria and has been thoroughly examined in the fields of cell biology and medical science. Xu’ group developed a mitochondrial-targeting fluorescent probe for the detection of fluorine in viable cells, which was denoted as **Mito-FP** (Xu et al., 2019). The probe could be successfully localized in the mitochondria and has been used for the imaging of fluorine in HeLa cells.

#### 2.2.4 Fluorescent Probes Detecting Mitochondrial Microenvironment

In addition to the mitochondrial morphology, several microenvironmental factors are regarded as the significant factors of the mitochondrial status, such as the mitochondrial pH value, polarity, and temperature. Normal polarization is necessary for cellular energy metabolism (Nemoto et al., 2000), and the appropriate pH value could also keep the normal membrane potential, which forces ATP generation and Ca^2+^ homeostasis regulation (Crompton and Heid, 1978).

**FIGURE 6:**
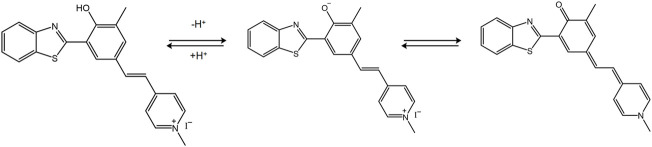
The pH sensing mechanism for the probe **HBTMP** (Gaoqing et al., 2020).

Gaoqing et al. (2020) designed the fluorescent probe **HBTMP** to detect the mitochondrial pH value. This probe emitted red fluorescence in acidic and neutral environments and blue fluorescence in alkaline environments (Figure 6). Furthermore, **HBTMP** exhibited improved photostability and lower cytotoxicity. The fluorescent image of the viable cells demonstrated that **HBTMP** could easily spread in the mitochondria and detect changes in the pH with high sensitivity. We concluded that **HBTMP** could be used to study the pH changes of the mitochondria in viable cells in a more efficient way. Li et al. (2019) reported a near-infrared fluorescence probe of hydroxy-L-lysine in 2019, denoted as **HXPI-P**. This probe was used to detect mitochondrial polarity changes through drug induction and starvation, which contributed to distinguish the differences in polarity between normal and cancer cells *via* ratio fluorescence imaging.

## 3 Fluorescent Probes Design Strategies for Targeting the Lysosomes

Lysosomes are important acidic organelles in eukaryotic cells. They involve more than 60 hydrolases and proteases and are considered to be the “digestive organs” of the cells. In addition, they can also participate in the regulation of the secretory function of cells. Lysosomes also contain various RSMs that participate in the corresponding biological reactions, such as ROS and metal cations. The visualization of RSMs in the lysosome plays an important role in understanding their mechanism of action and their therapeutic application in the treatment of various related diseases (Chen et al., 2021; Wang and Diao, 2022).

**FIGURE 7:**
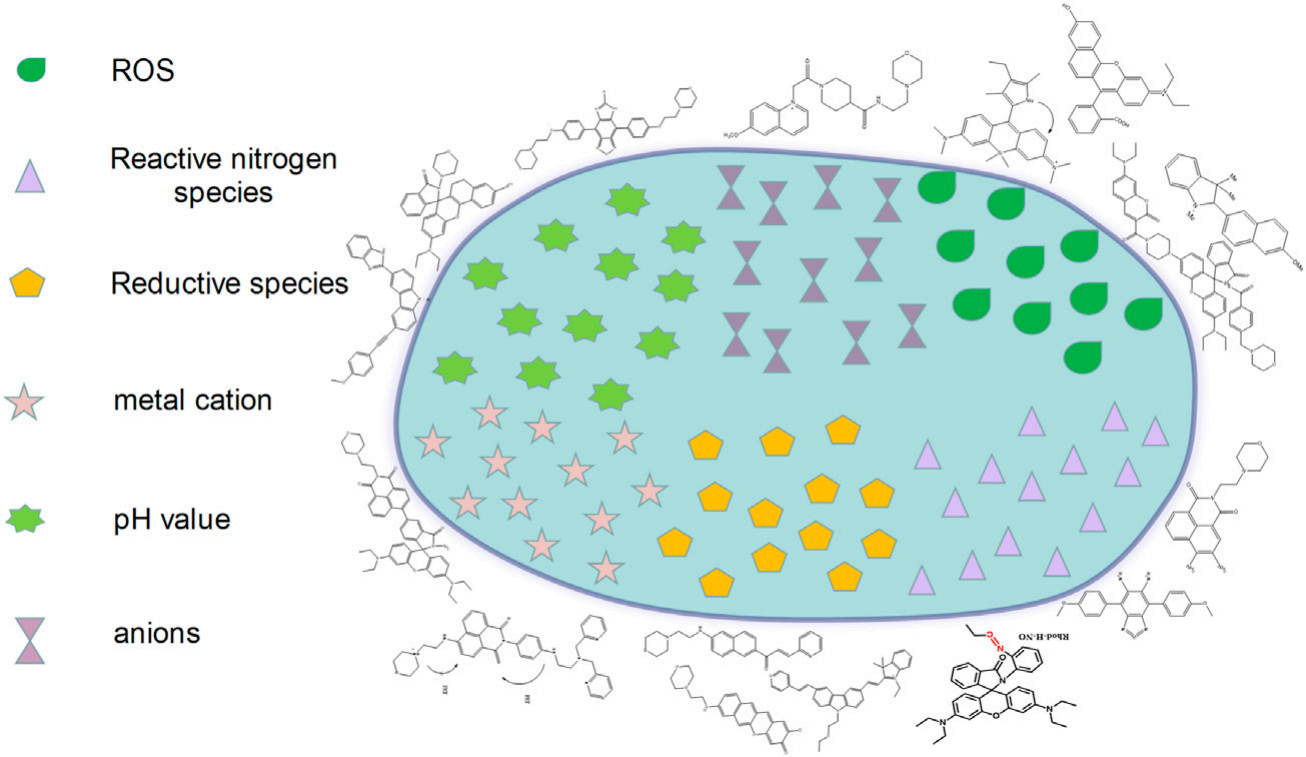
The design strategies of fluorescence probes targeting lysosomes.

A single functional fluorescent probe targeting lysosomes can enable imaging the lysosomes to detect their dynamic location and morphological changes and track their physiological processes (Qiu et al., 2020). However, the majority of the available commercial probes targeting lysosomes are amine-based compounds and exhibit certain limitations, such as lower specificity of their localization and reducing suitability for long time detection. To address these problems, researchers have carried out research on lysosomal probes (Figure 7).

### 3.1 Fluorescence Probes Detecting Reactive Oxygen Species in the Lysosome

**FIGURE 8:**
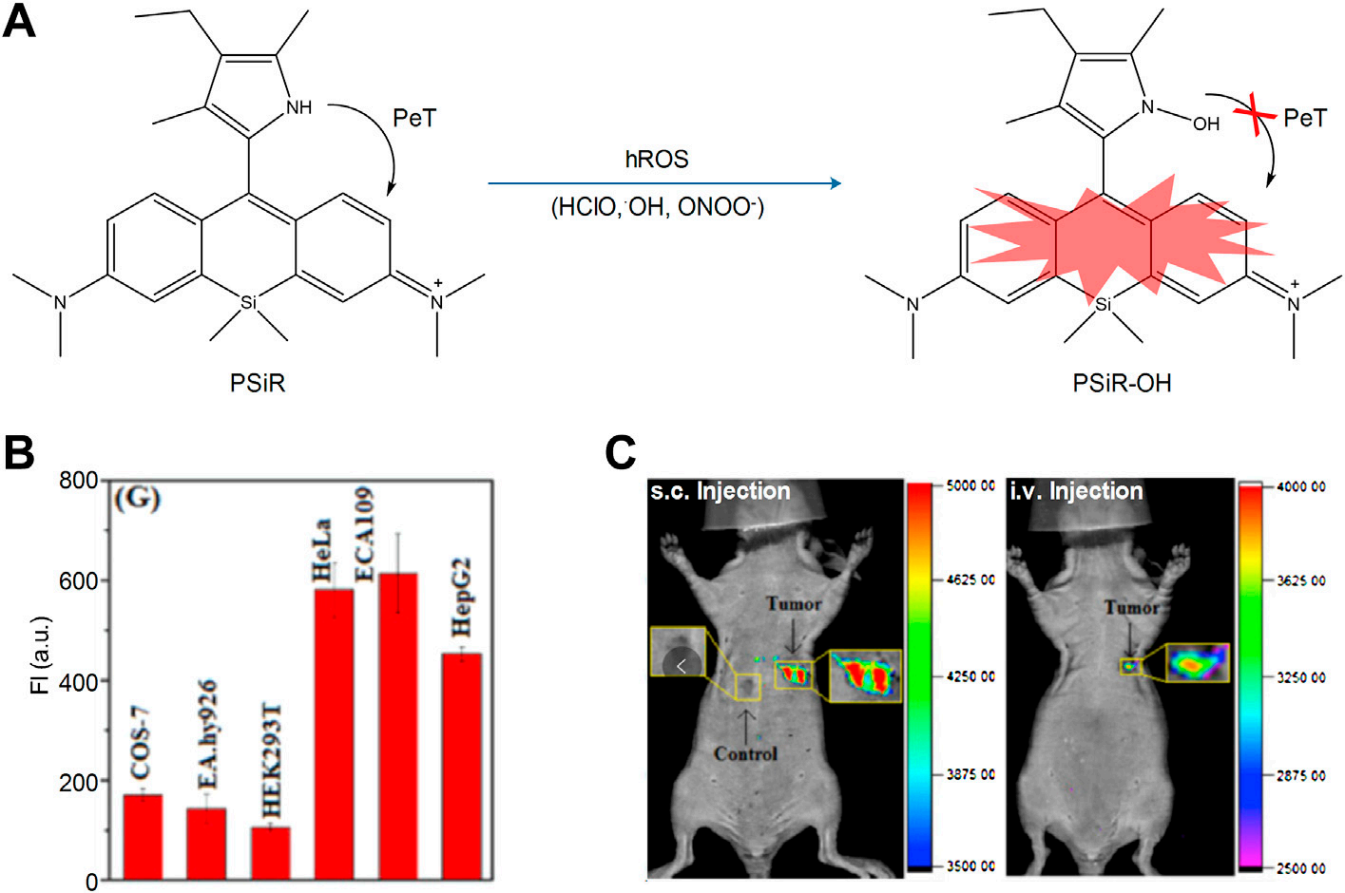
The sensing mechanisms of **PSiR** for hROS and imaging characterization (Zhang et al., 2017). **(A)** The sensing mechanisms of **PSiR** for hROS. **(B)** Average fluorescence intensity from images in various cells. **(C)** Imaging tumor using **PSiR** in tumor-bearing mouse with HeLa cells.

In cancer cells, the lysosomal content is high, and oncogene-driven transformation will alter the lysosomal membrane in cancer cells, which makes them more sensitive to lysosomal membrane permeability (LMP) and promotes tumor progression (Boya and Kroemer, 2008; Kallunki et al., 2013). Among the various intracellular stimuli (e.g., LMP), the ROS are the most closely related to lysosomal death (Vila et al., 2011; Melina-Theoni and Athanasios, 2014). In order to assess the ROS-related lysosomal cell death in cancer cells, Zhang et al. (2017) designed and synthesized a near-infrared fluorescent probe (**PSiR**) targeting the lysosomes, which could timely detect the generation of lysosomal ROS in cancer cells (Figure 8). The experimental results indicated that the probe exhibited strong resistance to photooxidation, fast reaction, and high selectivity and sensitivity. The anticancer drug β-lapachone (β-lap) could stimulate the generation of ROS in lysosomes, which was accompanied by a dose-dependent fluorescence enhancement. Due to its sensitivity in detecting ROS in cancer cells, the probe could distinguish normal cells from cancer cells according to specific images; it could also distinguish the presence of cancer cells in healthy tissues (Figure 8C).

The hydroxyl radical (•OH) is one of the most active free radicals noted in ROS, which can destroy various biomacromolecules. In order to detect the generation and function of lysosomal •OH in viable cells, Benitez-Martin et al. (2018) developed an active probe targeting lysosomes, denoted as **1-Red** (“off” state). HOCl is a type of ROS and lysosome is one of the main sources of HOCl. Therefore, the detection of lysosomal HOCl plays an important role in studying its biological process. In 2017, Yawei et al. (2017) designed and synthesized a pH-mediated lysosomal targeted fluorescent probe (**Lyso-HOCl**). The probe contained pH-sensitive phenol, which was used as its targeting group. A similar structure of rhodamine demonstrated a unique chlorination effect in an acidic environment, which could be used to specifically detect HOCl. Meng et al. (2019) developed the fluorescent probe **CR-Ly** using coumarin as a donor, rhodamine as the receptor, and morpholine as the lysosomal targeting group.

### 3.2 Fluorescent Probe Detection of Reactive Nitrogen Species in the Lysosome

**FIGURE 9:**
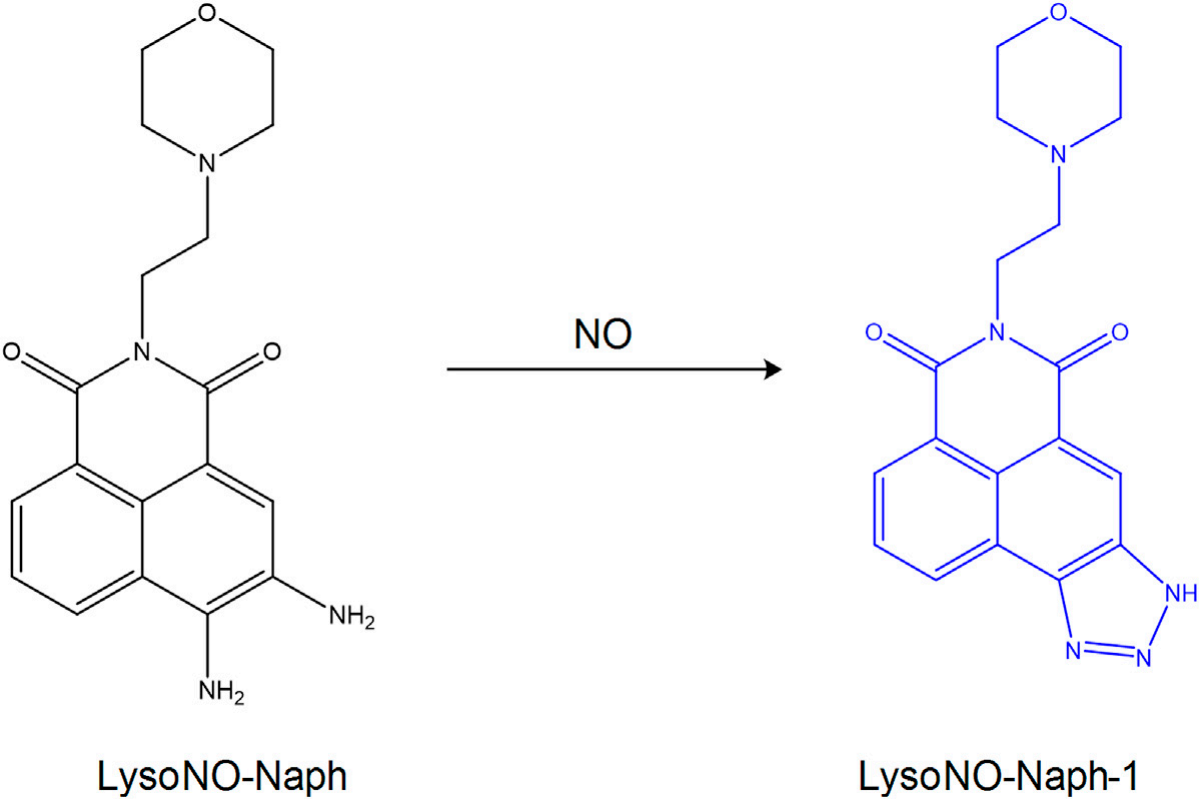
The reaction process of probe **LysoNO-Naph** with NO (Feng et al., 2016).

Nitric oxide (NO) plays an important role in the process of cell catabolism, whose quantity can influence lysosomal function. Abnormal NO can induce the development of cardiovascular and nervous system diseases. Therefore, the function of NO in lysosomes remains to be studied, which requires the design and development of ideal lysosomal targeting fluorescent probes detecting the change in the concentration of NO. Feng et al. (2016) synthesized the fluorescent probe **LysoNO-Naph** to detect NO in lysosomes on the basis of 1,8-naphthalimide (Figure 9). The probe was synthesized by using 4-(2-aminoethyl)-morpholine as a targeting group and o-phenylene-diamine as the reacting site of NO. And it could be used for lysosomal imaging. In addition, hemolysin-Naph exhibited higher selectivity and sensitivity for NO, indicating that this probe could be used to detect lysosomal NO successfully. In the same year, Yinhui et al. (2016) designed the pH-activated fluorescent probe **Rhod-H-NO** for detecting lysosomal NO levels. When the diameter of the nanoparticles was less than 200 nm, MSNs could enter into the lysosome, resulting in its successful tracking and imaging. Therefore, embedding **Rhode-H-NO** into the nanopore with MSNs can prevent it from being degraded, which leads to the accumulation of the probe in the lysosomes and the detection of NO. Fengyang et al. (2018) designed and synthesized the novel fluorescent probe **MBTD** in 2018. **MBTD** could specifically be used to image NO in lysosomes due to its large stroke shift and stable fluorescence; its D-A-D-structure probe exhibited improved photostability and higher NO selectivity.

### 3.3 Fluorescent Probe Detection of Reductive Species in Lysosomes

Hydrogen sulfide (H_2_S) is very important in various physiological processes. It can regulate cardiovascular and neuronal functions. H_2_S can also cause instability of the lysosomal membrane, which leads to autophagy and cell death. Low molecular weight mercaptans, such as cysteine (Cys) and glutathione (GSH), play an important role in the pathogenesis of related human diseases (Baocun et al., 2010). Therefore, the detection of the levels of H_2_S and mercaptans in the cells is very important for the identification of biological processes and the diagnosis and treatment of related diseases.

**FIGURE 10:**
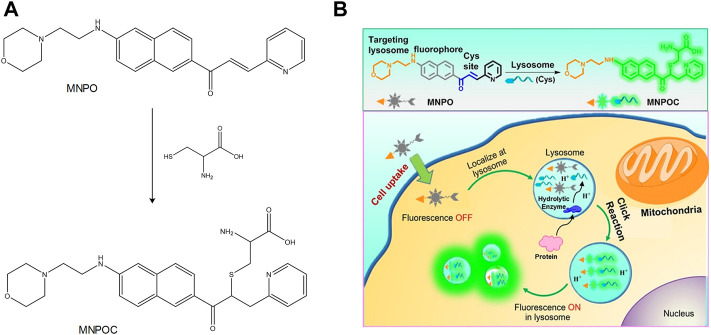
The action mechanism of **MNPO** with Cys in lysosomes (Long et al., 2019). **(A)** Proposed response mechanism of **MNPO** to Cys. **(B)** Schematic showing the general design of lysosome-targeting fluorogenic probe **MNPO** for Cys.

The hydrolysis of various proteins in the lysosome is closely related to mercaptan (T et al., 2000). In the lysosomes of the liver cells, Cys is a main stimulant for the degradation of albumin (Arunachalam et al., 2000). To investigate the function of Cys in lysosomes, Long et al. synthesized the TP probe **MNPO** (Figure 10) (Long et al., 2019). The probe was designed and synthesized with a naphthalene derivative as the fluorescent group, morpholine as the lysosomal targeting group, and α,β-unsaturated ketone as the action site of Cys. The introduction of the pyridine group into the molecule could improve water solubility and selectivity of Cys. It was found using specific experiments that the increase of Cys concentration increased the fluorescence intensity of the probe at 524 nm. Moreover, when the concentration range of Cys was 0–10 μM, the fluorescence intensity indicated a linear relationship with Cys. Therefore, the probe could be a useful tool to detect the dynamic changes of Cys in lysosomes. In addition, Tamima et al. (2020) developed a novel lysosome-targeted fluorescent probe **ABXO1** for the detection of the levels of Cys. Interestingly, the probe and cysteine adduct exhibited optimal two-photon absorption properties, which could achieve the two-photon imaging of lysosomal cysteine under excitation at near-infrared wavelengths.

H_2_S is an antioxidant that participates in various physiological reactions in the liver, spleen, and kidneys. Traditional lysosomal-targeting H_2_S fluorescent probes cannot provide adequate imaging of the lysosome with an open fluorescent signal; therefore, the design of fluorescent probes targeting lysosomal and mitochondrial H_2_S has high application value. Yong et al. (2016) designed and synthesized the TP probe **TP-PMVC**, which could be used to image lysosomal and mitochondrial H_2_S by using a dual channel. The probe used carbazole MVC as a TP platform. Since the pKa of pyridine was 5.0, it was used as the site of H^+^ and as the targeting unit of the lysosome. In addition, indole exhibited potent electrophilicity required for the generation of H_2_S. In an acidic environment, pyridine was protonated to produce red fluorescence for lysosomal imaging. By using specific experiments, it was shown that the fluorescent intensity was significantly improved under acidic conditions, while lysosomes and lysosomal H_2_S could be detected at 960 nm and 810 nm. In addition, Cai et al. (2018) synthesized the ratio fluorescent probe **SN-N3** for the detection of lysosomal H_2_S.

### 3.4 Fluorescent Probes Used for the Detection of the Metal Cation in Lysosomes

In recent years, a novel function of the lysosomes has been discovered which is the regulation of the steady state of the transition metals, such as copper and zinc (Blaby-Haas and Merchant, 2014). Various enzymes in the body can catalyze biochemical reactions by using specific transition metals. Therefore, lysosomes can maintain the metal steady state in cells by regulating the metal content and controlling its downstream signaling (Kurz et al., 2011; E. and S., 2014).

Copper is related to the activity of various essential enzymes in the body. When copper is absent, the activity of the enzymes dependent to copper will decrease leading to the occurrence of related diseases, such as Menkes syndrome (Bie et al., 2007; Tümer and Møller, 2010). In addition, excessive copper can also lead to cellular toxicity, affect lipid metabolism, and other biological processes, and subsequently lead to certain related diseases, such as Wilson’s disease (Seth et al., 2004). Therefore, in recent years, fluorescent probes detecting copper ions (Cu^2+^) in lysosomes have become a major focus of research investigation. In 2015, Mingguang et al. developed a lysosomal targeted Cu^2+^ fluorescent probe (**Lys-Cu**) with dual channel emission, which used rhodamine as a dye (Mingguang et al., 2015). The carbonyl oxygen atoms in 1,8-naphthalimide can combine with metal ions and can be used as a binding unit and a fluorescent group. During the process of photoinduced electron transfer, PET is inhibited following the combination of Cu^2+^ with 1,8-naphthalimide, leading to a significant enhancement of the fluorescent intensity at 440 nm and 580 nm. In addition, the probe exhibited lower cytotoxicity and higher affinity and could be used to image Cu^2+^ in lysosomes more efficiently. The hydrazone-containing pyrrole exhibited high affinity for Cu^2+^ and, in 2018, synthesized a hydrazone probe from sunitinib (Wu et al., 2018). The results indicated that when Cu^2+^ was combined with the probe, rhodamine was used to induce a ring opening reaction; pyrrole could then react with rhodamine resulting in the imaging of Cu^2+^ in lysosomes.

**FIGURE 11:**
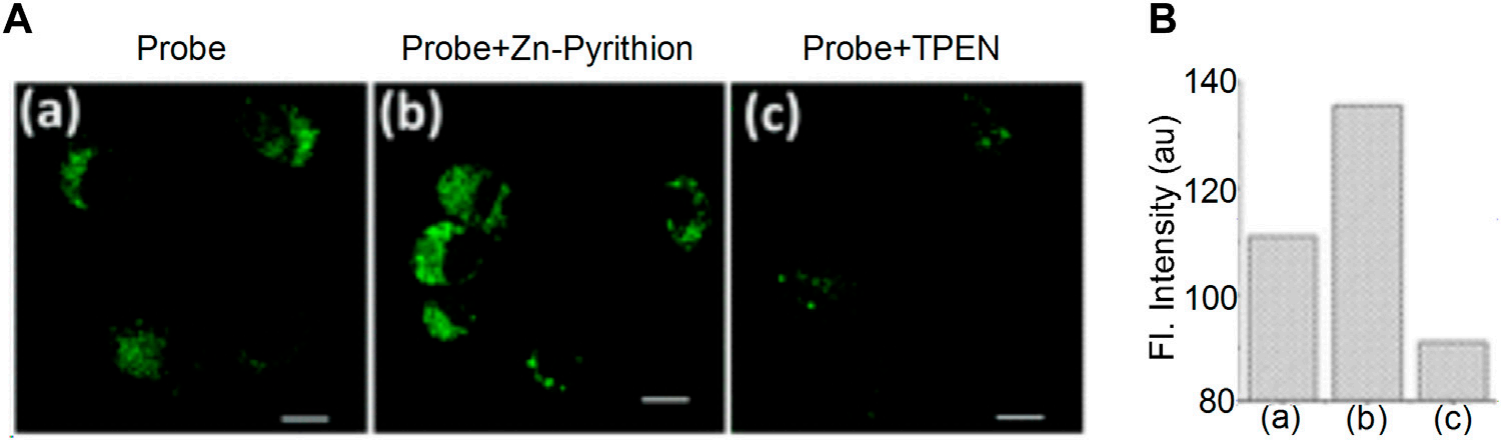
The fluorescence characteristics of probe (Hyo-Jun et al., 2016). **(A)** TPM imaging of Zn(ii) ions in live NIH 3T3 cells. **(B)** The substantial increase in fluorescence intensity after being uncubated with Zn-Pyrithion in the cells.

Zinc ions (Zn^2+^) are important metal ions involved in several biological reactions. They are closely related to lysosomal dysfunction and autophagy. The abnormal Zn^2+^ concentration levels can result in the development of human diseases, such as coronary heart disease (Heyu et al., 2021) and allergic inflammation (Masanobu et al., 2020). Currently, various fluorescent probes have been developed to detect Zn^2+^; however, only a few can be used to detect Zn^2+^ in lysosomes. In 2015, Hyo-Jun et al. introduced an N,N-di-(2-pyridyl)ethylenediamine (DPEN) group into the naphthalimide dye (Hyo-Jun et al., 2016). The oxygen atom in the carbonyl group of the imide was combined with Zn^2+^ and with the nitrogen atom in DPEN, subsequently a two-photon fluorescence probe was synthesized for the detection of Zn^2+^ in the lysosomes (Figure 11). It was found that the probe exhibited an optimal linear response to Zn^2+^; the fluorescent intensity of the probe was low following combination with Zn^2+^ when pH = 7.4. When pH = 4.5–5.5, the fluorescent intensity was significantly enhanced. The high sensitivity and affinity of the probe for Zn^2+^ was optimal for the detection of the dynamic changes of lysosomal Zn^2+^. In addition, Duan et al. designed the fluorescent probe **DR**, which was synthesized using N,N-bis(2-pyridylmethyl)ethylenediamine (BPEN) and morpholine as a ligand (Duan et al., 2019). When BPEN was connected with the fluorescent group by benzene on the imide, the probe could achieve high sensitivity to Zn^2+^. When the probe was combined with Zn^2+^, the fluorescent intensity increased at a pH range of 7.0–10.0 and was significantly increased when the pH range was 4.0–7.5, which indicated that the probe could be used to detect Zn^2+^ in lysosomes.

### 3.5 Fluorescent Probe Detecting Anions in Lysosomes

**FIGURE 12:**
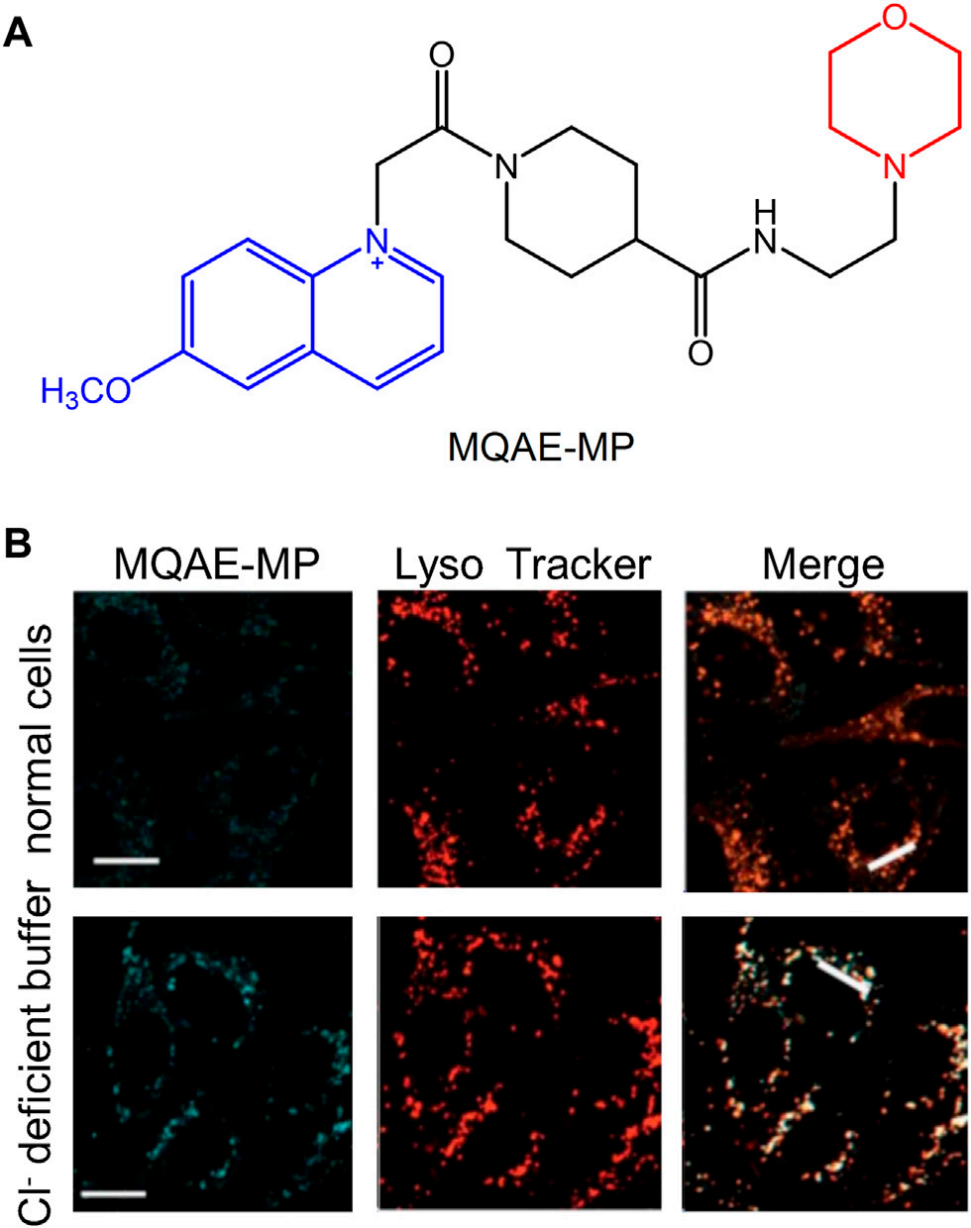
The structure and fluorescence characteristic of **MQAE-MP** (Sang-Hyun et al., 2019). **(A)** The chemical structure of **MQAE-MP**. **(B)** The detection of Cl^−^ ions in cells with **MQAE-MP**.

The maintenance of lysosomal pH is achieved by the synergy of the proton pump and the chloride channel. Moreover, the chloride (Cl^−^) ion plays an important role in the development of brain diseases, such as Alzheimer’s disease (Hui et al., 2017). Therefore, the development of fluorescent probes that can specifically detect Cl^−^ in lysosomes has become one of the major research hotspots in recent years. Sang-Hyun et al. (2019) developed the fluorescent probe **MQAE-MP** in 2019 to specifically detect lysosomal Cl^−^. It was found by using specific experiments that the concentration of Cl^−^ in lysosomes was decreased following treatment with the substances that could destroy their normal function. The concentration of Cl^−^ depended on whether **MQAE-MP** was used or not. Therefore, due to the targeting effect of the morpholine group, **MQAE-MP** was mainly accumulated in lysosomes and could be used to fully detect the levels of Cl^−^ in these organelles (Figure 12).

### 3.6 Fluorescent Probes Targeting pH Value in the Lysosome

**FIGURE 13:**
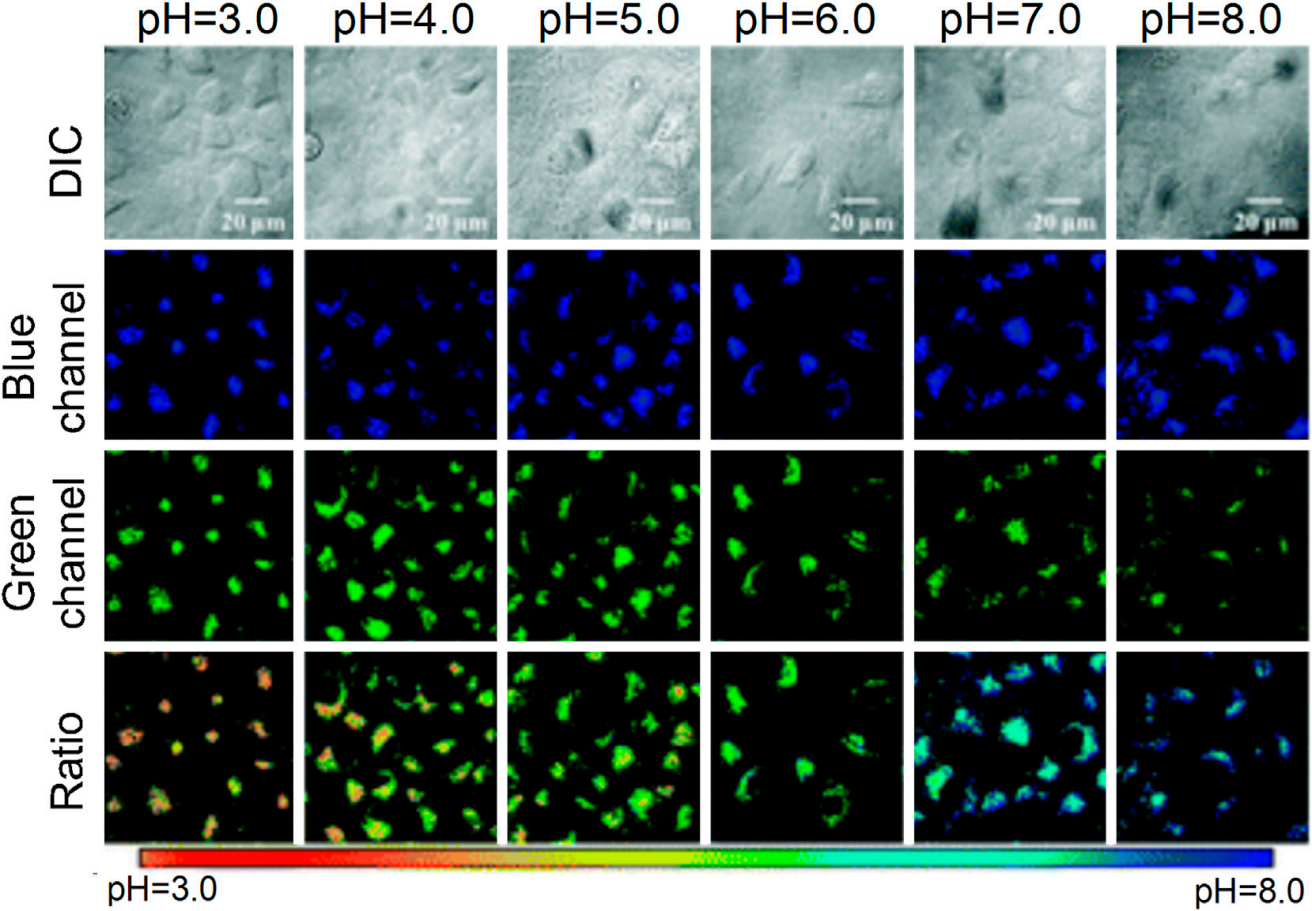
The action mechanism of **Lyso-MPCB** to lysosome pH value (Peng et al., 2019)

The normal pH of the lysosome is 4.5–5.5, which demonstrates weak acidity. An abnormal pH value can lead to corresponding changes in the cell function, and subsequently cause human-related diseases, such as cardiovascular and neurodegenerative diseases (Onyenwoke and Brenman, 2015). Therefore, the detection of the changes in lysosomal pH is important for understanding their biological functions in the related diseases. In the recent 10 years, various small-molecule fluorescent probes have been developed to detect the changes in the lysosomal pH (Wen et al., 2014; Jun et al., 2015; Yongkang et al., 2016; Ji-Ting et al., 2017). Despite these efforts, considerable work is required to improve their detection sensitivity. Peng et al. (2019) developed a two-photon fluorescence probe, which was sensitive to pH changes, and was denoted as **Lyso-MPCB** (Figure 13). The probe was equipped with the lysosome-located group morpholine, and it could monitor the pH value of the lysosome in real time. Moreover, it could specifically detect autophagy. **Lyso-MPCB** exhibited blue fluorescent emission at basic conditions and could emit green fluorescence at acidic conditions. The results indicated that the pKa value of the probe was 4.86, which was suitable for detecting the normal pH changes of the lysosomes (4.5–5.5). In addition, the ratio signal of the lysosome was linear with the pH when the range was 4.2–5.6. Therefore, the probe was a powerful tool for monitoring pH changes in lysosomes.

In addition, Lee et al. (2018) designed and synthesized a rhodamine pH fluorescent probe, which exhibited improved water solubility, higher quantum yield, and sensitivity and selectivity. The probe’s pKa value was 4.10, and therefore it was used to detect the acidic environment. Gong et al. (2019) designed a fluorescent probe detecting the pH changes in lysosomes, denoted as **Ly-HN2AM**, which used N-aminomorpholine as a closed loop switch and could react strongly under the range: pH = 4.79–6.07. The probe exhibited lower cytotoxicity and improved photostability and could be used to visualize the pH changes in lysosomes under physiological and pathological conditions. Lingling et al. (2020) synthesized a series of fluorescent probes in 2020 based on imidazole-benzothiadiazole compounds to detect the changes in pH of the lysosomes. The probe **MIBTAA** could react strongly in an acidic environment and exhibited higher sensitivity and selectivity for detecting pH changes.

## 4 Dual-Labeling Probes Targeting Lysosomes and Mitochondria

The lysosome is an acidic organelle that can decompose proteins, and mitochondria is considered the energy source of the cells. The dynamic changes and biological functions of lysosomes and mitochondria in viable cells can be detected simultaneously with fluorescence microscopy.

**FIGURE 14:**
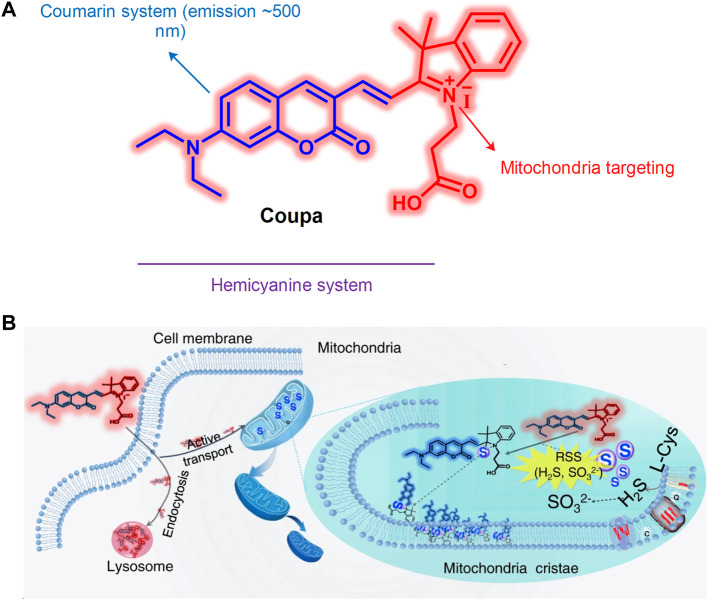
The structure and action mechanism of probe Coupa (Qixin et al., 2020). **(A)** The chemical structure of Coupa; **(B)** The mitochondria- and lysosome-staining mechanisms of Coupa.

The interaction between the mitochondria and lysosomes is an important biological process in eukaryotic cells. When their interaction is dysfunctional, certain neurodegenerative diseases develop (Nixon, 2013), such as cancer (Beth, 2007) and Parkinson’s disease (Burbulla et al., 2017). Therefore, an increased number of studies have visually assessed the interaction between mitochondria and lysosomes. Qixin et al. (2020) developed the hemicyanine fluorescent probe **Coupa** in 2020, which included a specific organelle-targeting ability (Figure 14). By using the technology of super-resolution illustrated microscopy (SIM), the **Coupa** dye could label mitochondria and lysosomes simultaneously and reveal the interaction between them by functional fluorescence conversion and co-localization. Staining with **Coupa** indicated that the local viscosity was increased, which was consistent with the biological characteristics of MLC. This may be the result of protein aggregation in the process of MLC. Therefore, the probe could be used efficiently to locate and track the interaction of the lysosomes with mitochondria in viable cells.

The abnormal viscosity of the mitochondria and lysosomes can lead to their dysfunction and eventually to the development of related diseases (Lu et al., 2013; Bochao et al., 2019). Wei et al. (2021) had developed a cyanine compound in 2020 for the detection of medium viscosity. The probe had near-infrared emission (650 nm) and improved sensitivity and selectivity. It could simultaneously target the lysosomes and mitochondria. In addition, this compound could distinguish normal cells and cancer cells by identifying the changes in viscosity; it could also detect and image the changes in the mitochondrial and lysosomal viscosity in HeLa cells.

SO_2_ is an important gaseous messenger, which plays a significant role in the induction of apoptosis in lysosomes and mitochondria. When SO_2_ exhibits abnormal levels, the N-acetylneuraminic acid present in lysosomes and mitochondria will redistribute, which exhibits adverse effects on the induction of apoptosis (Merkur’eva et al., 1981). Therefore, it is necessary to develop a fluorescent probe that can detect SO_2_ and simultaneously target the lysosomes and mitochondria. Kong et al. (2019) synthesized the novel fluorescent probe **DML-P** in 2019 by using a FRET-based method, which could simultaneously detect SO_2_ levels in lysosomes and mitochondria. The probe was the first double-targeted fluorescent probe that could simultaneously track SO_2_ in mitochondria and lysosomes. **DML-P** exhibited higher selectivity and stability. In addition, **DML-P** could detect cellular endogenous SO_2_ both in the single-photon and two-photon modes, indicating that it could become a powerful tool to study the action mechanism and relationship of the lysosomes and mitochondria.

Nitroreductase (NTR) plays an important role in human health, and the mitochondria and lysosomes are its main sources (Yang et al., 2018; Klockow et al., 2020). Therefore, the development of fluorescent probes for the detection of NTR in mitochondria and lysosomes has become one of the main research hotspots. Sha et al. (2020) developed a dye with aromatic azonia and benzo(e)indol anion skeleton in 2020. On the basis of this dye, the authors of the study synthesized a probe containing 2-ethyl-5-nitrofuran or 4-nitrobenzoyl moiety to detect NTR. The probe emitted in the near infrared. It could target lysosomes and mitochondria simultaneously and exhibited higher sensitivity to NTR. This probe could detect and image lysosomal and mitochondrial NTR in HeLa cells. Therefore, it became the first fluorescent compound which could simultaneously detect lysosomal and mitochondrial NTR.

## 5 Conclusion and Outlook

Small-molecule fluorescent probes located in the mitochondria and lysosomes have been used to detect and image RSMs in recent years. This area has become a research hotspot and involves the identification of fluorescent probes targeting the mitochondria and lysosomes. These probes exhibit specific targeting capabilities and have become a popular research tool in the field of biology, pharmacy, and clinical medicine. Among them, two-photo fluorescent probes have been widely included in the study of lysosomal and mitochondrial targeted fluorescent probes due to the advantages of high resolution and long-time observation. At present, various fluorescent probes have been designed and synthesized to simultaneously target the mitochondria and lysosomes, which provides important tools for the study of RSMs and their mechanisms of action.

According to the present review, the probes detecting lysosomal and mitochondrial RSMs fit the following characteristics: 1) higher selectivity toward certain RSMs compared with other RSMs noted in the organelles; 2) higher quantum yield and longer emission wavelength; 3) higher photostability of product with probe and RSMs; 4) improved biocompatibility. Currently, the emission wavelength of the fluorescent group in certain probes remains short and subject to interference; therefore, the research and development of fluorescent probes require further studies to be completed.

At present, various mitochondrial and lysosomal targeted fluorescent probes have enabled the dynamic monitoring and imaging of RSMs, and certain probes have been used in the diagnosis and treatment of human-related diseases, which will become one of the important research directions in the future.
